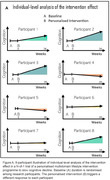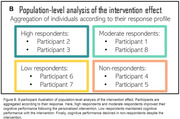# The effectiveness of a personalised multidomain lifestyle intervention programme to slow cognitive decline: study protocol for a series of randomised open‐label N‐of‐1 trials

**DOI:** 10.1002/alz.088066

**Published:** 2025-01-09

**Authors:** Wayne Freeman Chong, Nagaendran Kandiah, Mohammed Adnan Azam

**Affiliations:** ^1^ Lee Kong Chian School of Medicine, Nanyang Technological University, Singapore Singapore; ^2^ GeroPsych Consultants Pte Ltd, Singapore, Singapore Singapore; ^3^ Nanyang Technological University, Singapore, Singapore, Singapore Singapore

## Abstract

**Background:**

Multidomain lifestyle interventions for dementia risk reduction have been developed and trialled because reversible lifestyle factors have been shown to contribute to the onset and progression of dementia. A recent review and meta‐analysis confirmed small beneficial effects of such interventions on cognitive performance. To enhance the effectiveness of these interventions, we have developed and incorporated personalisation approaches. This study aims to investigate the effectiveness of a personalised multidomain lifestyle intervention programme in slowing cognitive decline.

**Method:**

A series of randomised open‐label trials in a single research participant (N‐of‐1 trials) will be conducted with 100 participants who are 50 years and over, and with mild cognitive impairment (MCI) and cerebral small vessel disease (CSVD). These participants will be recruited from an ongoing panel study that evaluates global and specific cognition, demographic, behavioural and vascular risk characteristics, lifestyle habits, blood‐based biomarkers and brain structure, among community dwelling Singapore residents. With a multiple baseline design situated within a 12‐week period, the baseline duration for each participant will vary systematically and randomly (Figure A). Baseline measurements should appear stable before each participant starts the intervention. The intervention is a 24‐week programme in which a study clinician reviews available baseline data, discloses the MCI and CSVD classification, provides standard cognitive health education electronically, communicates a dementia risk score via the use of the Cardiovascular Risk Factors, Aging, and Incidence of Dementia Risk Score App, discusses and recommends lifestyle activities involving some combination of a tailored exercise regimen, gamified digital cognitive activities, and nutritional guidance, with intensity personalized according to the baseline vascular risk characteristics and CSVD burden of each participant, and provides behavioural counselling utilising cognitive‐behavioural and motivational strategies. Adherence to the lifestyle activities will be assessed fortnightly using wearable trackers, in‐game cognitive assessments, and a digital food diary respectively.

**Result:**

Global cognition at 24 weeks and at 1‐, 3‐ and 6‐month follow‐up will be compared with baseline global cognition for each participant. Data aggregation could uncover response characteristics across individuals (Figure B).

**Conclusion:**

Meta‐analyses would provide high quality evidence of the intervention’s effectiveness at the individual and the population levels.